# The Relationship Between Guilt and Help-Seeking in Parent-Adult Child Dyads: A Daily Diary Study

**DOI:** 10.1093/geroni/igaf122.3060

**Published:** 2025-12-31

**Authors:** Zewen Huang, Da Jiang

**Affiliations:** The Education University of Hong Kong, Hong Kong, China (People’s Republic); The Education University of Hong Kong, Hong Kong, China (People’s Republic)

## Abstract

Help-seeking behaviors play a crucial role in maintaining the quality of parent-adult child relationships. However, little is known about the psychological factors that influence whether parents and adult children seek help from each other. Guilt, an emotional response to perceived wrongdoing or unmet expectations, may discourage help-seeking, as individuals may view it as a burden on family or a sign of dependency. This study examined the within-person association between guilt and help-seeking and whether this relationship differs by family role (i.e., parent vs. adult child) using a 14-day daily diary survey from 103 parent-adult child dyads (Mage (parents) = 57.18, SD = 6.70, 68.93% women; Mage (adult children) = 22.67, SD = 4.94, 84.47% women). Guilt was measured as participants’ anticipated guilt when imagining seeking help from their partner on that day, and help-seeking was assessed by asking whether they sought any of six types of support (e.g., companionship, talking about daily events, emotional support, practical support, giving advice, and financial assistance) that day. Multilevel modeling revealed that on days when parents and adult children felt guiltier about seeking help, they were less likely to do so (β = -.04, p < .05). Such relationships did not differ by family role (i.e., as a parent or a child; β = .06, p = .096). These findings highlight that individuals who experience guilt may avoid seeking support even from close family members. Understanding this dynamic provides insights into potential interventions to increase help behavior and promote healthier parent-adult child relationships.

